# Sickle cell vaso-occlusive crisis: it’s a gut feeling

**DOI:** 10.1186/s12967-016-1092-5

**Published:** 2016-12-01

**Authors:** Seah H. Lim, Loren Fast, Alison Morris

**Affiliations:** 1 Division of Hematology and Oncology, Rhode Island Hospital/Brown University Warren Alpert Medical School, Room 140 APC Building, 593 Eddy Street, Providence, RI USA; 2 Division of Pulmonary, Allergy, and Critical Care Medicine, University of Pittsburgh Medical Center, Pittsburgh, PA USA

## Abstract

Insights in the pathogenesis of vaso-occlusive crisis in patients with sickle cell disease have changed significantly in the last decade. Various laboratory and clinical evidence have provided support to the pivotal role of activated neutrophils in this process. A recent study in murine sickle cell disease indicated that the intestinal microbiota is responsible for regulating the number of aged neutrophils, a subset of neutrophils that are overly activated. Reduction of these neutrophils in vivo protected the mice from fatal TNFα-induced vaso-occlusive crisis. In this paper, we discuss the reasons why patients with sickle cell disease may have an abnormal intestinal microbiota and how this could contribute to the development of vaso-occlusive crisis. We also highlight the recent interest in studying the intestinal microbiota of patients with sickle cell disease and suggest that the next therapeutic approach for these patients may well be in the manipulation of the intestinal microbiota to restore the individual’s microbial landscape.

The human intestine is colonized by thousands of different microbial species. The gut microbiota plays a central role in microbial homeostasis, regulation of metabolism, and immune tolerance. A novel method involving the use of mass DNA sequencing targeting the 16S ribosomal RNA has allowed a comprehensive evaluation of the microbiome. This has led to clinical findings implicating dysbiosis in an assortment of systemic conditions [1–5].

Sickle cell disease (SCD) is a classic inherited disorder of a nucleotide mutation, causing a single amino acid substitution from glutamic acid to valine at position 6 on the β-globin subunit. This substitution leads to changes in the physical properties of the globin chain. During physiological stresses, e.g. infections, hypoxia, and dehydration, hemoglobin S (HbS) polymerizes, causing erythrocytes to adopt a sickle morphology and become more rigid, with the consequent expression of various cellular adhesion molecules, e.g. α4β1 [6] and Lu/BCAM [7] that may facilitate the physical interaction of the sickle erythrocytes with endothelium. However, the interaction between sickle erythrocytes and the endothelium is unlikely to be the central issue in vaso-occlusive crisis (VOC) because there is a lack of correlation between the number of sickle erythrocytes observed in the peripheral blood and VOC symptoms or severity.

Neutrophils play a pivotal role in initiating VOC. Their importance has been highlighted in various laboratory studies and clinical observations [8–10]. In a mouse model of VOC, instead of attaching to endothelium, sickle erythrocytes more commonly adhered to activated neutrophils [8]. SCD patients have higher levels of soluble CD62L, a serum marker of neutrophil activation [9]. Neutrophils from SCD patients also express higher levels of activation molecules, e.g. CD64 [9] and CD11b/CD18 [10], that mediate their adherence to endothelium. These immobilized neutrophils may act as niduses for sickle erythrocytes to attach to [11] and cause VOC. Hematopoietic growth factor administration to a SCD patient, who was a donor for hematopoietic progenitor cell transplant, upregulated the activation molecules and led to fatal consequence [12]. Other observations that support a link of VOC with neutrophils include the correlation between higher neutrophil counts and severity of the disease [13], and between responses to hydroxyurea, irrespective of the hemoglobin F (HbF) levels, and decreased neutrophil counts [14]. Inflammatory cytokines [15–19] and monocytes also exacerbate VOC [20]. VOC, therefore, occurs as a result of the interplay between sickle erythrocytes, activated neutrophils, inflammatory cytokines, and endothelium, with activated neutrophils being the central players.

So what is the main source of stimulus responsible for inducing neutrophil activation in SCD patients? A novel theory that the intestinal microbiome is critical in triggering VOC has recently been proposed [21]. Rapidly evolving understanding of the importance of the gut microbiome implicates changes in the composition and function of gut microbial communities in not only gastrointestinal diseases, but also in an assortment of systemic conditions [1–5]. SCD may be the latest to join this list based on experimental evidence in animal models as well as clinical characteristics of SCD and VOC that support a theoretical framework of microbial involvement.

Limited data currently exist in animal models. A study in SCD mice demonstrated that the intestinal microbiota was responsible for regulating the number of VOC-promoting neutrophils [21]. Depletion of the intestinal microbiota with prolonged courses of combination antibiotics was associated with a corresponding reduction in the number of circulating aged neutrophils, a subset of neutrophils that are overly activated, and an improvement in the clinical outcome from TNFα-induced VOC. This function of the intestinal microbiota in regulating aged neutrophils appeared to operate through Toll-like receptor (TLR)-mediated and myeloid differentiation factor 88 (Myd88)-mediated signaling mechanisms [21]. This work, therefore, highlights the importance of the intestinal microbiota in regulating ageing of neutrophils in SCD and that microbiota depletion reduces severity of experimental VOC.

Additional support for involvement of the microbiota in SCD stems from the observation that SCD patients have higher numbers of baseline activated neutrophils [9, 10] compared to healthy controls, and SCD patients receiving penicillin for prophylaxis have fewer circulating activated neutrophils [21]. As suggested by the mouse study [21], if intestinal microbes are the main source of activators of circulating neutrophils in SCD patients, it follows that there must be abnormalities in either the integrity of the intestinal barriers in SCD or a chronic disequilibrium of the intestinal microbiota, or both. The intestinal barrier separates the intestinal lumen from the inner host. It consists of mechanical elements (mucus, epithelial layer), humoral elements (defensins, IgA), immunologic elements (lymphocytes, innate immune cells), muscular and neurological elements. Failure of the protective intestinal barrier results in recurrent translocation of intestinal bacteria that can stimulate inflammation without causing overt infection or that can lead to bacteremia/septicemia. We hypothesize that both the intestinal barrier and the ability of the mucosa to support a normal intestinal microbiota in SCD patient are compromised, due to segmental or global subclinical intestinal ischemia that occurs as a result of VOC in the splanchnic vasculature. The compromised intestinal barrier renders SCD patients more susceptible to recurrent translocation by bacterial inoculums not high enough to cause overt infections, but sufficient to activate neutrophils. Furthermore, as a result of the mucosa’s inability to support a normal microbiota, the normal intestinal microbiome is disturbed. In some patients, this disturbance may be worsened by certain diet, medications, or lifestyle that results in the production of metabolites that stimulate neutrophil activation. Both bacterial translocation and intestinal dysbiosis likely contribute to the higher levels of circulating activated neutrophils observed in SCD patients.

The need for further research into the role of microbiota in mediating diseases was highlighted in May 2016 when the White House announced the establishment of the National Microbiome Initiative. The Minority Coalition for Precision Medicine (MCPM), which has an interest in the interplay between intestinal microbiota and VOC, is involved in this initiative and aims to collect the stool samples from 500 donors with SCD and sickle trait. To confirm the hypothesis that the microbiota is critical in VOC, this study and others are needed to determine if an abnormal intestinal microbiome correlates with increased numbers of circulating activated neutrophils and more frequent VOC. Studies will also be needed to confirm the importance of specific bacteria or their function in SCD and VOC, and additional mechanistic animal studies will be needed to confirm associations seen in humans. Confirmation of the microbiome hypothesis of SCD will open up new avenues of therapeutic approach to establish and maintain a normal intestinal microbiota for patients with SCD such as use of pre- and pro-biotics, manipulation of intestinal microbial species using fecal microbiota transplant, and therapies to reduce bacterial burden and translocation.

In conclusion, VOC may now join the ever-expanding list of pathologic states regulated by intestinal microbiota. We expect that there will be an explosion of activities in this area in the next few years. The next therapeutic approach for SCD patients might well be via manipulation of the intestinal microbiota. For sickle cell VOC, the answer may lie in the intestine!

## Abbrevations

SCD: sickle cell disease
VOC: vaso-occlusive crisis
